# Hydroquinine Inhibits the Growth of Multidrug-Resistant *Pseudomonas aeruginosa* via the Suppression of the Arginine Deiminase Pathway Genes

**DOI:** 10.3390/ijms241813914

**Published:** 2023-09-10

**Authors:** Sattaporn Weawsiangsang, Nontaporn Rattanachak, Touchkanin Jongjitvimol, Theerasak Jaifoo, Pensri Charoensit, Jarupa Viyoch, Sukunya Ross, Gareth M. Ross, Robert A. Baldock, Jirapas Jongjitwimol

**Affiliations:** 1 Biomedical Sciences Program, Faculty of Allied Health Sciences, Naresuan University, Phitsanulok 65000, Thailand; sattapornw63@nu.ac.th; 2 Biology Program, Faculty of Science and Technology, Pibulsongkram Rajabhat University, Phitsanulok 65000, Thailand; nontaporn.r@psru.ac.th (N.R.); touchkanin@psru.ac.th (T.J.); 3 Master of Science in Medical Technology, Faculty of Allied Health Sciences, Naresuan University, Phitsanulok 65000, Thailand; teerasak.lab@gmail.com; 4 Department of Pharmaceutical Technology, Faculty of Pharmaceutical Sciences, Naresuan University, Phitsanulok 65000, Thailand; pensric@nu.ac.th (P.C.); jarupav@nu.ac.th (J.V.); 5 Center of Excellence for Innovation in Chemistry, Naresuan University, Phitsanulok 65000, Thailand; 6 Biopolymer Group, Department of Chemistry, Faculty of Science, Naresuan University, Phitsanulok 65000, Thailand; sukunyaj@nu.ac.th (S.R.); gareth@nu.ac.th (G.M.R.); 7 Centre of Excellence in Biomaterials, Faculty of Science, Naresuan University, Phitsanulok 65000, Thailand; 8 School of Pharmacy and Biomedical Sciences, Faculty of Science and Health, University of Portsmouth, Portsmouth PO1 2DT, UK; 9 Department of Medical Technology, Faculty of Allied Health Sciences, Naresuan University, Phitsanulok 65000, Thailand

**Keywords:** arginine deiminase pathway, drug resistance, hydroquinine, *Pseudomonas aeruginosa*

## Abstract

Hydroquinine has antimicrobial potential with demonstrated activity against several bacteria, including multidrug-resistant (MDR) *P. aeruginosa* reference strains. Despite this, there is limited evidence confirming the antibacterial activity of hydroquinine against clinical isolates and the underlying mechanism of action. Here, we aimed to investigate the antibacterial effect of hydroquinine in clinical *P. aeruginosa* strains using phenotypic antimicrobial susceptibility testing and synergistic testing. In addition, we examined the potential inhibitory mechanisms against MDR *P. aeruginosa* isolates using informatic-driven molecular docking analysis in combination with RT-qPCR. We uncovered that hydroquinine inhibits and kills clinical *P. aeruginosa* at 2.50 mg/mL (MIC) and 5.00 mg/mL (MBC), respectively. Hydroquinine also showed partial synergistic effects with ceftazidime against clinical MDR *P. aeruginosa* strains. Using SwissDock, we identified potential interactions between arginine deiminase (ADI)-pathway-related proteins and hydroquinine. Furthermore, using RT-qPCR, we found that hydroquinine directly affects the mRNA expression of *arc* operon. We demonstrated that the ADI-related genes, including the arginine/ornithine antiporter (*arcD*) and the three enzymes (arginine deiminase (*arcA*), ornithine transcarbamylase (*arcB*), and carbamate kinase (*arcC*)), were significantly downregulated at a half MIC of hydroquinine. This study is the first report that the ADI-related proteins are potential molecular targets for the inhibitory effect of hydroquinine against clinically isolated MDR *P. aeruginosa* strains.

## 1. Introduction

Antimicrobial resistance against clinical pathogens poses a significant global threat to public health. This challenge arises due to the diminished effectiveness of treating microbial infections, ultimately leading to increased human mortality [1]. *Pseudomonas aeruginosa*, especially its multidrug-resistant strains, has been classified as one of the most concerning pathogens by the World Health Organization [1]. It is an opportunistic infection often affecting hospitalized patients with limited effective therapeutic options [2]. *P. aeruginosa* is able to resist the effects of antibiotics through various mechanisms, including beta-lactamase production, reduced outer membrane permeability, efflux pump overexpression, the production of aminoglycoside-modifying enzymes, and target modification [3,4]. These mechanisms confer resistance to many types of antibiotics, such as aminoglycosides, fluoroquinolones, beta-lactams, chloramphenicol, etc., and therefore create the potential for multidrug resistance (MDR) [5]. According to the National Nosocomial Infections Surveillance System, *P. aeruginosa* was the second most common microorganism isolated in nosocomial pneumonia (17% of cases) [6]. It was the third most common pathogen isolated in patients with surgical site infections and urinary tract infections (11% of cases) [6]. Overall, *P. aeruginosa* is the fifth most common microorganism isolated from all specimens in nosocomial infection patients (about 9% of cases) [7]. Therefore, the rise of antibiotic-resistant *P. aeruginosa* infections has prompted researchers to focus on novel anti-infective agents.

Alternative new agents derived from natural products offer another promising option for combating the growth of MDR *P. aeruginosa* [8]. Natural bioactive compounds extracted from medicinal plants (e.g., flavonoids, alkaloids, and terpenoids) have several antibacterial mechanisms and anti-infective mechanisms; for example, inhibiting quorum sensing (QS) molecules [8,9]. As a result, natural compounds may possess effective antimicrobial properties [8,9]. Many natural products have been documented as potential antimicrobial agents to directly inhibit the growth of pathogenic microorganisms and/or act synergistically to potentiate the effect of other antibiotics [8].

Hydroquinine is an organic compound that has been shown to possess antimicrobial properties [10]. Kraikongjit et al. (2018) [11] and Jongjitvimol et al. (2020) [12] have previously demonstrated that ethanolic nest entrances extracts from *Tetrigona apicalis* exhibit antibacterial, antifungal, and anti-proliferative activities [11,12]. Interestingly, one of the key chemical compounds found from the extracts was the polyphenol, hydroquinine. In the Netherlands, hydroquinine has been used as a medicinal medication to relieve nocturnal cramps [13]. Concerning the potential safety of using hydroquinine, the compound may also reduce light-brown patches on the skin and skin discolorations associated with pregnancy, although further investigation is required to determine its potential clinical use. Despite this, hydroquinine has been shown to have anti-malarial and demelanizing activities [14]. 

Rattanachak et al. (2022) uncovered the antibacterial activities of hydroquinine; they identified its potential in inhibiting and killing both Gram-positive and Gram-negative bacteria, including *Staphylococcus aureus*, *Enterobacter *cloacae**, *Escherichia coli*, *Klebsiella pneumoniae*, and, in particular, *P. aeruginosa* [15]. The study revealed that hydroquinine had the highest minimum inhibitory concentration (MIC) values (2.50 mg/mL) against the drug-sensitive (DS) *P. aeruginosa* ATCC 27853. In contrast, hydroquinine showed a lower MIC (1.25 mg/mL) against the MDR *P. aeruginosa* BAA-2108 strain [15]. According to this finding, hydroquinine is more effective against the MDR strain than the drug-sensitive strain [15]. Jongjitwimol and Baldock (2023) proposed that hydroquinine has potential as an antimicrobial agent, highlighting the ability of the compound to target MDR strains of *P. aeruginosa* [16]. Rattanachak et al. (2022) mechanistically investigated the inhibitory effects of ½ MIC hydroquinine through the evaluation of the global transcripts of *P. aeruginosa* using RNA sequencing with high-throughput transcriptomic analysis [17]. They found that hydroquinine inhibited several virulence factors, such as downregulating flagellar-related genes, which govern bacterial motility, and reducing QS-related genes, affecting the reduction of pyocyanin production and biofilm formation [17]. Moreover, several additional genes were identified as having up- or downregulated expression with high confidences (Figure S1). The observation of significant downregulation of four genes from the arginine deiminase (ADI) pathway (namely, *arcA, arcB, arcC,* and *arcD*) in response to hydroquinine treatment is intriguing (Table S1) [17]. For example, the arginine/ornithine antiporter (AOA) encoded by the *arcD* gene was the most downregulated in the transcriptomic analysis by a −4.24 Log_2_-fold change in response to hydroquinine. Consistently, the other arginine deiminase (ADI)-pathway-related genes, including *arcA* (arginine deiminase; ADI)*, arcB* (ornithine transcarbamylase; OTC)*,* and *arcC* (carbamate kinase; CK) were also significantly downregulated in response to hydroquinine, by −3.85, −3.32, and −3.41 Log_2_-fold changes, respectively [17].

Among bacteria, the ADI pathway is widely distributed, where it is frequently a major source of bacterial energy [18]. This pathway provides some energy in *P. aeruginosa* under anaerobic conditions because of the lack of oxygen or terminal electron acceptors. In *P. aeruginosa*, the expression of the functional genes (*arcA, arcB,* and *arcC*) is preceded by *arcD*, which encodes the arginine–ornithine antiporter [19]. The expression of these genes and the function of their protein products are critical for the intracellular ATP supply and, consequently, dysfunction of these genes affects bacterial cell growth [19,20,21,22].

As the *arc* genes are downregulated when treated with hydroquinine compared to the controls, we hypothesized whether the attenuation of the ADI pathway is potentially one of the mechanisms of action used by hydroquinine to inhibit the bacterial growth of *P. aeruginosa* strains. Here, we investigated the antibacterial effect of hydroquinine in vitro against clinical *P. aeruginosa* strains using phenotypic antimicrobial susceptibility and synergistic testing. Furthermore, we validated the differential expression of the ADI-pathway-related genes using quantitative reverse transcription polymerase chain reaction (RT-qPCR) and investigated the potential for the direct binding of hydroquinine to ADI pathway factors using molecular docking analysis on solved X-ray crystallographic and AlphaFold predicted structures. Uncovering the molecular target or targets of hydroquinine in *P. aeruginosa* will be an important step and may suggest future targets for drug design and/or help to pre-empt any future mechanism of resistance.

## 2. Results

### 2.1. Phenotypic Characterization of Antibiotic Susceptibility Profiles

The antibiotic susceptibility profiles of *P. aeruginosa* from the six clinical isolates and one reference strain were phenotypically investigated with several antibiotic classes. We demonstrated that all *P. aeruginosa* strains were sensitive to amikacin. In contrast, certain clinical *P. aeruginosa* isolates were resistant to specific anti-pseudomonal drugs. For example, resistance to ceftazidime was identified in three clinical isolates; namely, PA-S2, PA-S4, and PA-S5.

Two clinical *P. aeruginosa* isolates, PA-S3 and PA-S6, were sensitive to all antibiotic agents tested, which was comparable to the reference drug-sensitive strain (PA-27853). PA-S1 was sensitive to all drugs except, partially, levofloxacin. PA-S2 was only resistant to ceftazidime. In contrast, two clinical *P. aeruginosa* isolates, PA-S4 and PA-S5, were defined as MDR as they showed resistance to ≥1 agent in ≥3 antimicrobial classes. Particularly, the PA-S4 strain resisted ceftazidime, piperacillin/tazobactam, and cefoperazone/sulbactam, whereas the PA-S5 strain resisted all agents in the carbapenems group (doripenem, imipenem, and meropenem), and it also resisted the cephalosporins group (ceftazidime and cefepime), fluoroquinolones group (ciprofloxacin and levofloxacin), as well as cefoperazone/sulbactam. A summary of the results obtained for all strains tested with all antimicrobial agents is provided in Table 1.

### 2.2. Hydroquinine Inhibits and Kills MDR P. aeruginosa Isolated from Clinical Samples

There were six representatives of clinical *P. aeruginosa* strains isolated from a blood sample (PA-S1), two samples of sputum (PA-S5 and PA-S6), and three samples of pus (PA-S2, PA-S3, and PA-S4). Hydroquinine could inhibit and kill all clinical isolates at MIC and minimum bactericidal concentration (MBC) values of 2.50 and 5.00 mg/mL, respectively. Comparable to the clinical strains, *P. aeruginosa* ATCC 27853 was inhibited and killed by the same concentrations of hydroquinine (Table 2).

According to the phenotypic characterization of the antibiotic susceptibility profiles, PA-S4 and PA-S5, which were isolated from pus isolated from eye infection and sputum, respectively, were identified as clinical *P. aeruginosa* MDR strains. Importantly, these clinical MDR strains were still inhibited and killed by the same concentration of hydroquinine. The MDR strains showed significant resistance to ceftazidime, with high MIC (≥32 ug/mL) (Table 1). 

### 2.3. Hydroquinine Demonstrates Partial Synergistic Effect with Ceftazidime against Clinical MDR P. aeruginosa Strains 

In combination, the MICs of hydroquinine and ceftazidime were reduced compared to those of the individual agents by two to eight times in both strains (PA-S4 and PA-S5) as shown in Table 3. The MIC values of ceftazidime were decreased by two-fold (MIC/2) when combined with the hydroquinine treatment. According to the fractional inhibitory concentration index (∑FICI), hydroquinine had notable partial synergistic effects with ceftazidime (∑FICI of 0.750 and 0.625 against clinical MDR *P. aeruginosa* strains PA-S4 and PA-S5, respectively).

### 2.4. Molecular Docking Simulations Reveal the Potential for Interaction between Hydroquinine and ADI-Pathway-Related Target Proteins

**Figure 1:**
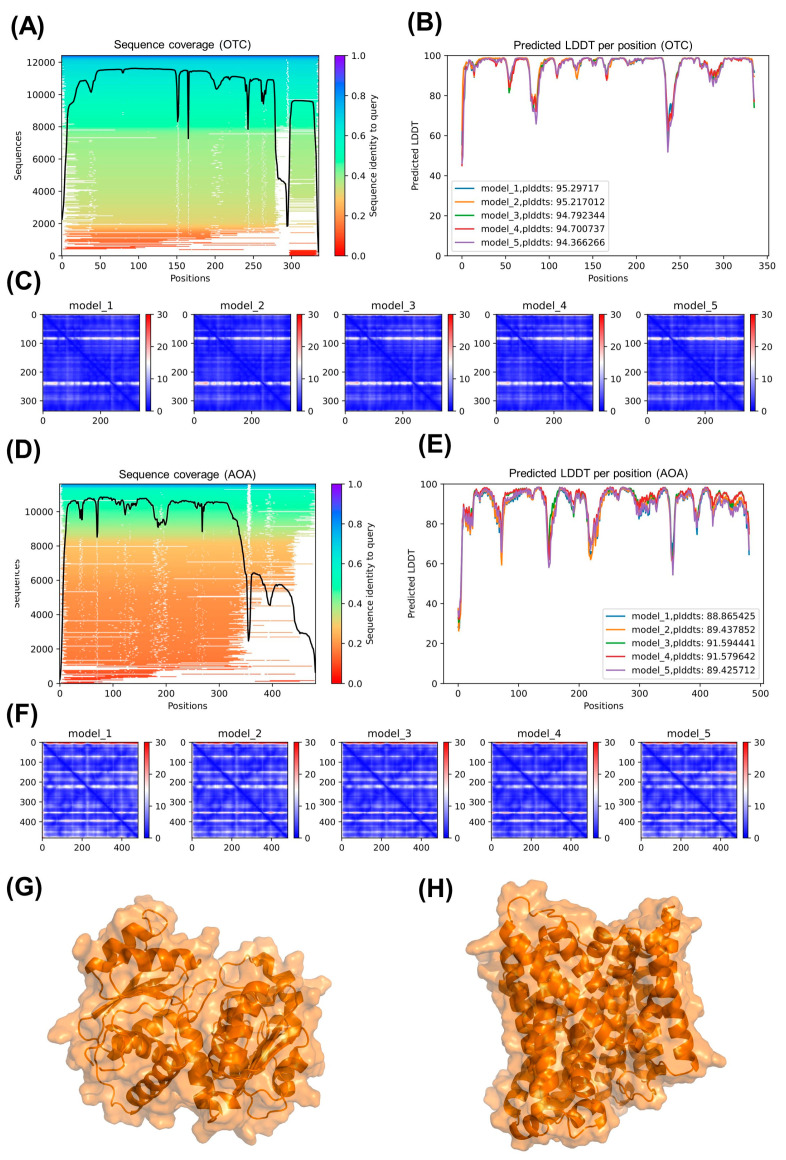
AlphaFold predicted structures of ornithine transcarbamylase (OTC) and arginine/ornithine antiporter (AOA) from *P. aeruginosa*. (**A**) shows a multiple sequence alignment heatmap showing coverage of the query sequences for OTC. The black line indicates the relative coverage. (**B**) pLDDT (prediction of performance on the local distance difference test) plot shows model confidence per position of the amino acid sequence for each of the five models generated (pLDDT > 90, high confidence). (**C**) shows heatmaps of the predicted aligned error for each residue for the five models generated for OTC. Blue sections represent regions with low predicted error, whereas red indicates regions of higher predicted error. (**D**–**F**) show the equivalent plots for AOA. (**G**,**H**) show the final models of OTC and AOA, respectively, following Amber relaxation of the optimal model. Images were generated in PyMol molecular modeling software.

To investigate the potential for interactions between protein products of ADI-related genes and hydroquinine, we used molecular docking simulations to identify and prioritize potential binding targets and sites. Molecular docking of hydroquinine to each of the ADI-pathway-related proteins was performed using the SwissDock web server. Where possible, the solved X-ray crystal structures were used for the docking simulations, as was the case for ADI (encoded by *arcA*) and CK (encoded by *arcC*) (1RXX and 8CRV, respectively; available from: wwpdb.org) [23,24,25]. Unfortunately, as a solved crystal structure for *P. aeruginosa*, OTC (encoded by *arcB*), and AOA (encoded by *arcD*), we used Deepmind’s AlphaFold protein structure prediction run from the GitHub site [26]. The monomeric structures were predicted from primary amino acid sequences of the proteins. Figure 1 shows the multiple sequence alignment of the sequences queried (Figure 1A,D) for OTC and AOA, respectively. The confidence scores for the predicted structures (Figure 1B,E) for OTC and AOA show the predicted score on the local distance difference test (pLDDT) per residue (pLDDT scores > 90 indicate a high confidence prediction; scores between 70 and 90 indicate good performance on backbone structures; scores between 50 and 70 indicate low confidence; and pLDDT scores < 50 are unreliable or potentially indicate unstructured/intrinsically disordered regions). Five models each were predicted for OTC and AOA and evaluated for pLDDT per residue as well as the predicted aligned error (PAE). The predicted aligned errors for each residue against others in the five models for OTC and AOA are shown in the heatmaps in Figure 1C,F, respectively. The highest confidence model was relaxed using the Amber relaxation model to reduce the incidence of steric clashes by side chains by allowing rotation of the side chains. The final optimal relaxed models for OTC (model 1) and AOA (model 3) are shown in Figure 1G,H, respectively. 

**Figure 2:**
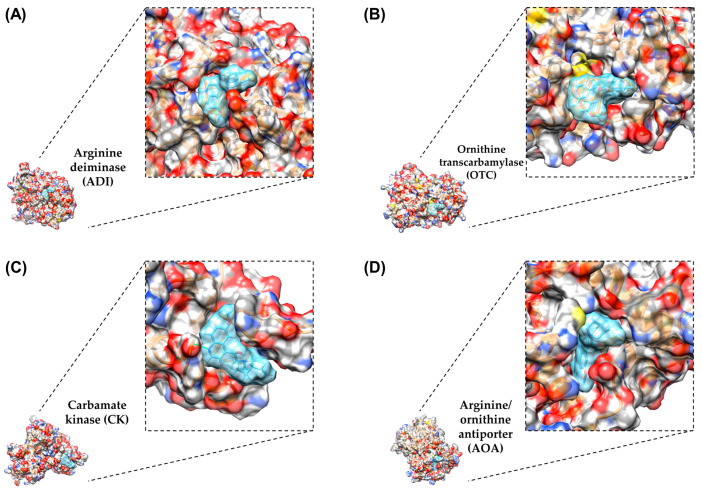
The electrostatic mapping of molecular docked hydroquinine to the four predicted target proteins: (**A**) arginine deiminase, (**B**) ornithine transcarbamylase, (**C**) carbamate kinase, and (**D**) arginine/ornithine antiporter. The 3D model representation of the hydroquinine ligand (cyan color) and the ribbon proteins (orange color) was visualized using UCSF-Chimera.

Using the solved protein crystal structures for ADI and CK alongside the AlphaFold predicted structures of OTC and AOA, the molecular docking demonstrates the possibility of an interaction between the ADI-pathway-related target proteins, including ADI, OTC, CK, and AOA, and hydroquinine based on the predicted binding free energy (ΔG) value. ADI, OTC, CK, and AOA had ΔG binding energies of −7.5571, −7.1706, −7.6305, and −7.7443 kcal/mol, respectively (Table 4). It is notable that the predicted binding free energy (ΔG) value of hydroquinine binding with AOA was the lowest value compared to that of the target proteins (suggesting a more favourable interaction), potentially correlating with the prioritization of targets from the transcriptomic data. Protein–ligand docking simulations were visualized with electrostatic interactions shown in red and blue (indicating negative and positive potential, respectively) using UCSF-Chimera. The models shown suggest that hydroquinine potentially interacts with the binding pockets of all target proteins (Figure 2). 

### 2.5. Hydroquinine Inhibits P. aeruginosa Growth through Decreased Expression of ADI-Pathway-Related Genes

**Figure 3:**
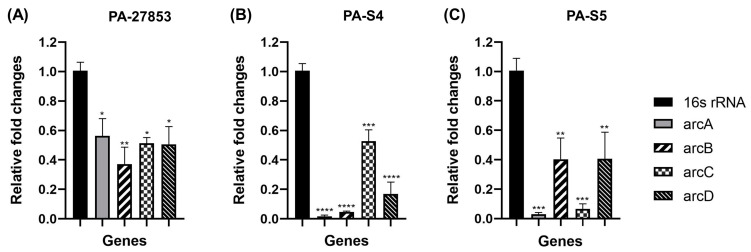
The relative expression levels of the ADI-pathway-related genes treated with hydroquinine at 1.25 mg/mL for 1 h, in (**A**) *P. aeruginosa* ATCC 27853, (**B**) PA-S4, and (**C**) PA-S5, compared to the corresponding untreated controls. The asterisk *, **, *** and **** symbols are *p* < 0.05, *p* < 0.01, *p* < 0.001, and *p* < 0.0001, respectively. The data are presented as mean ± SD.

To validate the altered expression of the four *arc* genes in response to hydroquinine, the drug-sensitive (PA-27853) and clinical MDR (PA-S4 and PA-S5) *P. aeruginosa* strains were investigated further to represent reference and clinical isolates, respectively. Using RT-qPCR, we identified that a ½ MIC (1.25 mg/mL) of hydroquinine reduces *arcDABC* gene expression in PA-27853, PA-S4, and PA-S5 (Figure 3). Specifically, in PA-27853, the RT-qPCR results showed statistically significant decreases in the mRNA expression of the *arcA, arcB, arcC,* and *arcD* genes to 0.56 ± 0.20, 0.37 ± 0.20, 0.51 ± 0.07, and 0.50 ± 0.21-fold, respectively (Figure 3A). For the clinical MDR PA-S4 strain, the relative expression levels of the *arcA, arcB, arcC,* and *arcD* genes were downregulated to 0.02 ± 0.01, 0.05 ± 0.01, 0.53 ± 0.13, and 0.17± 0.14-fold, respectively (Figure 3B), whereas those in PA-S5 were statistically downregulated to 0.03 ± 0.02, 0.40 ± 0.34, 0.07 ± 0.06, and 0.41 ± 0.31-fold, respectively (Figure 3C).

## 3. Discussion

In the present study, we present strong evidence that hydroquinine possesses bacteriostatic and bactericidal properties against clinical isolates of *P. aeruginosa*. This is consistent with the results of hydroquinine against *P. aeruginosa* reference strains [15]. Hypothetically, clinical strains from difference sources, including blood, pus, and sputum, are expected to adapt themselves in challenging conditions more than reference strains because of their virulence factors (e.g., adhesion, invasion, biofilm formation, etc.) [27]. However, our results showed that hydroquinine inhibits *P. aeruginosa* at a MIC of 2.50 mg/mL and kills the bacterium at an MBC of 5.00 mg/mL for all the *P. aeruginosa* strains tested, including at least two MDR strains (Table 2). This is consistent with a previous study in which the same concentrations of hydroquinine inhibited bacterial growth and killing of a *P. aeruginosa* reference strain (ATCC 27853) [15]. According to phenotypical antibiotic susceptibility profiles, only PA-S4 and PA-S5 strains are defined as clinical MDR pathogens due to showing resistance to more than three antimicrobial classes. Interestingly, these clinical MDR strains showed significant resistance to ceftazidime, with a high MIC (≥32 ug/mL). We therefore hypothesized that hydroquinine might show a synergistic effect with ceftazidime against the MDR PA-S4 and PA-S5 strains. Ceftazidime, a third-generation cephalosporin, is one of the β-lactam antimicrobials, inhibiting cell wall synthesis leading to bacterial cell death [28,29]. However, the MDR strains have many antibiotic resistance mechanisms, such as beta-lactamase production, reduced outer membrane permeability, efflux pump overexpression, production of aminoglycoside-modifying enzymes, and target modification [3,4,5]. These two MDR strains, therefore, were chosen to examine the combined activity of ceftazidime alone and with hydroquinine. The combination treatment demonstrated that hydroquinine had a partial synergistic effect with ceftazidime (Table 3). The indication that hydroquinine could potentiate the activity of current anti-pseudomonal drugs against clinical MDR *P. aeruginosa* strains is, potentially, a significant finding.

According to the work of Rattanachak et al. (2022), there were several changes in the transcriptomes of *P. aeruginosa* ATCC 27853 when treated with a hydroquinine concentration of 1.25 mg/mL for 1 h. This study revealed 157 upregulated genes and 97 downregulated genes in response to the treatment [17]. Several differentially regulated genes have already been investigated relating to virulence factors, such as quorum sensing and flagella assembly. All of the 254 DEGs were re-assessed, and the top 15 significantly up- and downregulated genes are presented in the heatmap (Figure S1). Interestingly, four of the top fifteen downregulated genes were integral to the ADI pathway; namely, *arcA, arcB, arcC,* and *arcD* genes (Table S1). We therefore hypothesized whether the sub-MIC of hydroquinine treatment is sufficient to reduce the ADI-pathway-related gene expression in *P. aeruginosa* strains. 

To further validate these findings, mRNA expression levels of all four genes were quantified using RT-qPCR. Following sub-MIC hydroquinine treatment, the expression levels of *arcA, arcB, arcC,* and *arcD* genes were significantly decreased. It also appears that the *arc* gene expression levels in clinical MDR *P. aeruginosa* isolates are affected to a greater extent than those in *P. aeruginosa* ATCC 27853. Interestingly, we also found that the mRNA levels of *arcA* in clinical MDR strains, PA-S4 and PA-S5, had the greatest downregulation of gene expression when compared with other genes (Figure 3). However, the reason why hydroquinine attenuates *arc* gene expression in MDR strains is still unknown, although this will be the subject of further study. 

**Figure 4:**
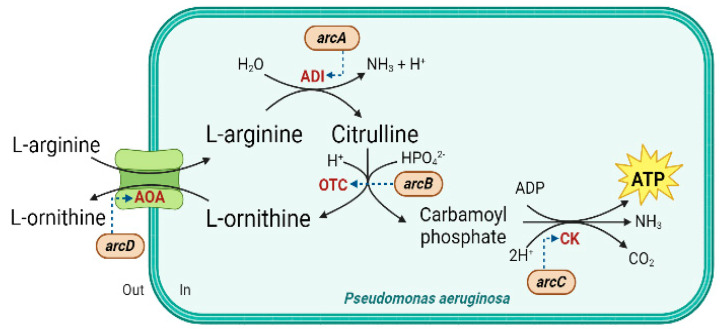
The schematic of the arginine deiminase (ADI) pathway. AOA, arginine/ornithine antiporter; ADI, arginine deiminase; OTC, ornithine transcarbamylase; CK, carbamate kinase.

Among the four *arc* genes, the *arcD* gene encodes a key membrane-bound transporter, the arginine/ornithine antiporter (AOA), which exchanges one molecule of L-arginine with one molecule of L-ornithine. In contrast, the *arcA*, *arcB,* and *arcC* genes encode the three important enzymes (namely, arginine deiminase (ADI), ornithine transcarbamylase (OTC), and carbamate kinase (CK), respectively) (Figure 4) [19,20,21,22].

The ADI pathway is conserved in a variety of bacteria, including *P. aeruginosa*. It produces one ATP mole from every mole of L-arginine consumed through three metabolic conversion steps. The equations of each step are presented as follows.

(1) $$\text{L-arginine}+H_{2}O\overset{\mathrm{ADI}}{\to }\text{L-citrulline}+{\mathrm{NH}}_{3}$$

(2) $$\text{L-citrulline}+P_{i}\overset{\mathrm{OTC}}{\to }\text{L-ornithine}+\text{Carbamoyl-P}$$

(3) $$\text{Carbamoyl-P}+\mathrm{ADP}\overset{\mathrm{CK}}{\to }\mathrm{ATP}+{\mathrm{NH}}_{3}+{\mathrm{CO}}_{2}$$

Firstly, L-arginine is catalyzed by ADI and converted into L-citrulline and ammonia. Secondly, the carbamoyl part of L-citrulline is then converted by OTC, resulting in L-ornithine and carbamoyl phosphate. The cluster of carbamoyl phosphate, which is a major metabolite in the ADI crossroad for both L-arginine catabolism and anabolism, is required for the initial step of pyrimidine biosynthesis [30]. Subsequently, the phosphate moiety of carbamoyl phosphate is transferred to adenosine diphosphate (ADP) by the CK, yielding ATP, ammonia, and CO_2_ [19,20,21,22]. Overall, the ADI pathway allows the bacterial cells to sustain ATP production in the oxygen-dependent respiratory chain from carbamoyl phosphate [31] and produce ATP in anoxic environments [22,32]. 

According to the RT-qPCR results in this study, hydroquinine reduced the expression levels of the *arc* genes in the *P. aeruginosa* strains of both DS and MDR. Interestingly, the ADI pathway may be one of the potential mechanisms by which hydroquinine inhibits *P. aeruginosa* growth. Here, we proposed that hydroquinine may directly bind one of the proteins, ADI, while the expression levels of other proteins in the ADI pathway are also decreased. Validation of the downregulation of genes by RT-qPCR reveals that *arcA* expression is reduced to the greatest extent, particularly the MDR *P. aeruginosa* strain. The effect of inhibiting ADI pathway activity would suppress the subsequent generation of ATP via L-citrulline and carbamoyl phosphate metabolism. ATP production in *P. aeruginosa* is required for cell division and bacterial growth [33]. We hypothesize that hydroquinine may disturb metabolic energy generation by interrupting ATP production, which consequently reduces bacterial cell growth. Evidence for this is provided by Sandra et al. (2021), who demonstrated that arginine fermentation provided sufficient energy levels for anaerobic growth of the meat-spoiling *Pseudomonas* strains (e.g., *Pseudomonas lundensis, Pseudomonas weihenstephanensis*, and *Pseudomonas fragi*) [32]. Furthermore, anaerobically produced ATP through the ADI pathway is required to maintain the membrane potential as well as to promote the motility of *P. aeruginosa* [34,35]. The ADI pathway normally supports protection from acidic stresses through intracellular ammonia production and from being involved in the microbial pathogenicity [18,36]. As a result, it is possible that hydroquinine may not only disturb the energy production effect but also affect ammonia generation via the ADI pathway. When ammonia production is decreased, *P. aeruginosa* would be unable to tolerate this with acid stress and pH homeostasis, which is critical for survival in acidic conditions. It has been shown that intracellular acidification may affect bacterial growth and cell viability [18]. 

Further investigation into the potential targeting of ADI pathway proteins by hydroquinine focused on molecular docking simulation. The molecular docking results are mainly estimated by the minimum binding free energy (ΔG) between the ligand and the target [37,38]. The ΔG values of hydroquinine bound to AOA, ADI, CK, and OTC indicated the strongest potential binding energy with AOA. Furthermore, it is consistent with the transcriptomic results that *arcD* transcripts were downregulated most by hydroquinine. Therefore, we suggest that hydroquinine is most likely to interfere with AOA, resulting in the repression of other downstream proteins in the ADI pathway. For example, if AOA is affected by hydroquinine, L-arginine intake would likely also be reduced. In general, L-arginine in *P. aeruginosa* is an essential molecule in the regulation of biofilm formation [39]. This supports the previous findings by Rattanachak et al. [17] that hydroquinine could suppress QS-related gene expression, reduce virulence factor production, and impair biofilm formation in *P. aeruginosa* [17]. For another possible reason, hydroquinine may affect the production of carbamoyl phosphate, which is required for pyrimidine production [30]. Theoretically, pyrimidine biosynthesis provides thymine, uracil, and cytosine nucleotides, which play a crucial role in DNA and RNA replication [40]. Therefore, we suggest that hydroquinine might have other specific mechanisms, like certain antibiotics, as a DNA synthesis inhibitor. This is supported by the structural evidence of a cinchona alkaloid (e.g., quinine, which has a DNA-binding capacity) possibly inhibiting the transcription and translation processes [41]. So, we propose that hydroquinine might have a synergistic effect with other antibiotics via the inhibitions of the ADI pathway and DNA synthesis, as well as their own mechanisms of anti-pseudomonal drugs.

We suggest that the ADI pathway could be a key target for hydroquinine, and this pathway plays an important role in the antibacterial mechanism of hydroquinine against the MDR *P. aeruginosa* strain. Further work will seek to establish the exact molecular target of hydroquinine in eliciting its antimicrobial effects. Furthermore, we will also look to identify whether hydroquinine could be used to enhance the effectiveness of existing antibiotics and pre-empt future mechanisms of resistance to hydroquinine. 

## 4. Materials and Methods

### 4.1. Hydroquinine Preparation

Hydroquinine powder (CAS No. 522-66-7) was purchased from Sigma-Aldrich. The working solution of hydroquinine (20 mg/mL) was prepared in 25% DMSO in Mueller–Hinton broth (MHB, Oxoid, Basingstoke, UK). Indicated concentrations of hydroquinine were diluted in MHB to achieve the required initial concentration [15,17]. The concentration range of hydroquinine employed in this study was based on published studies by Rattanachak et al. (2022) [15,17].

### 4.2. Strains and Cultivation of Pseudomonas aeruginosa 

The six *P. aeruginosa* clinical strains (PAS1-6) were kindly provided by Kamphaeng Phet Hospital. These strains were isolated from specimens, such as blood, pus, and sputum, obtained from hospitalized patients at Kamphaeng Phet Hospital, Kamphaeng Phet, Thailand in 2022. A reference strain of drug-sensitive (DS) *P. aeruginosa* ATCC 27853 (PA-27853) was purchased from the American Type Culture Collection (ATCC). 

A colony of each bacterium tested in this study was cultured on 5% sheep blood agar (BioMérieux, Inc. Hazelwood, MO, USA) at 35 ± 2 °C for 18–24 h. For subculturing, isolated bacterial colonies were re-streaked on the Mueller–Hinton Agar (MHA, Oxoid, Basingstoke, UK) and then incubated at 35 ± 2 °C for 24 h. The turbidity of the inoculum was adjusted to 0.5 McFarland standard, approximately 1–2 × 10^8^ CFU/mL [15,17].

### 4.3. Antibiotic Susceptibility Testing of P. aeruginosa Strains

For all *P. aeruginosa* strains, the antibiotic susceptibility testing (AST) was performed by the agar dilution method with the automatic Vitek^®^2 compact device (BioMérieux, Inc. Hazelwood, MO, USA). Briefly, 40 µL of the prepared inoculum was added into the AST test card, containing premeasured dried amounts of a specific antibiotic combined with the culture medium. The growth turbidity was measured every 15 min for a maximum incubation of 18 h at 35.5 ± 1 °C by the wavelength of 660 nm in each well at 16 different positions and repeated in triplicate. The antibiotics and interpretation used in this study were undertaken from the Clinical and Laboratory Standards Institute (CLSI) document M100 recommendation [42]. The used antibiotics were divided into six classes, namely: (i) aminoglycosides (amikacin), (ii) carbapenems (doripenem, imipenem, meropenem), (iii) cephalosporins (ceftazidime, cefepime), (iv) fluoroquinolones (ciprofloxacin and levofloxacin), (v) penicillins with beta-lactamase inhibitors (piperacillin/tazobactam), and (vi) cephalosporins with beta-lactamase inhibitor (cefoperazone/sulbactam). For interpretation, the MDR definition is the resistance to three or more antimicrobial classes [43,44,45,46].

### 4.4. Antibacterial Activity of Hydroquinine by Broth Microdilution Method

The antibacterial activity of hydroquinine against all *P. aeruginosa* strains was determined from the minimum inhibitory concentration (MIC) using broth microdilution assay with some modification of the CLSI guideline M07-A9 [47]. The minimum bactericidal concentration (MBC) was performed, as described in the previous study [15]. For the determination of MICs, the different dilutions of hydroquinine (ranging from 0.25 to 10 mg/mL) in MHB were added to 96-well microtiter plates. For the quality control (QC), *P. aeruginosa* ATCC 27853 was tested with ciprofloxacin (CIP), while all strains were cultured in MHB containing DMSO as vehicle controls. The positive and negative controls were wells containing only MHB with and without inoculum, respectively. Then, 10 µL of bacterial inoculum was added to each well to achieve the final inoculum concentration of approximately 5 × 10^4^ CFU/well. Then, the plates were incubated at 35 ± 2 °C for 16–20 h. The MIC values were the lowest concentrations of hydroquinine that inhibited bacterial growth, which can be seen clearly by unaided eyes. After the MIC was recorded, the MBCs were determined. Briefly, 10 µL of each tested well was dropped to MHA plates and then incubated at 35 ± 2 °C for 24 h. The MBC value is the lowest concentration without colonies growth [15].

### 4.5. Synergistic Activity Using Broth Microdilution Checkerboard Method

The synergistic effect of hydroquinine and ceftazidime was tested against clinical MDR *P. aeruginosa* strains by reading the MIC value of a single indicated agent and a combination of the indicated agents and then calculating the fractional inhibitory concentration index (∑FICI). The broth microdilution checkerboard technique was performed using a 96-well plate, modified from Fratini et al. (2016) and Cheypratub et al. (2018) [48,49]. Briefly, the final concentrations of ceftazidime and hydroquinine in combination were between 2×MIC and MIC/64. After that, 10 µL of the final inoculum (5 × 10^4^ CFU/well) was added into each well. Moreover, the vehicle and the positive and negative controls were performed similarly to the previous method. Then, the plates were incubated at 35 ± 2 °C for 16–20 h. The turbidity was observed by unaided eyes to determine the MIC values. The synergistic effect was determined by the fractional inhibitory concentration (FIC) index value, resulting from the changes in the MIC value [48,49]. The ∑FICI values were calculated from the formula ∑FICI = (MIC _HQ + antibiotic_ / MIC _HQ_) + (MIC _antibiotic + HQ_ /MIC _antibiotic_) and interpreted in terms of synergy <0.5; partial synergy, 0.5–0.75; additive effect, 0.76–1.0; indifference, >1.0; and antagonism was defined as ∑FICI > 4.0, respectively [50].

### 4.6. AlphaFold Protein Structure Prediction

AlphaFold protein structure prediction was performed by running AlphaFold v2 opensource code run via docker (available from: https://github.com/deepmind/alphafold (accessed on 10 July 2023)) [26]. Fasta files of protein sequences of OTC and AOA were generated and run. Five models were generated for each inputted fasta sequence. The quality of the predictions was evaluated for the predicted score on the local distance difference test (pLDDT) and the predicted aligned error. The optimal models for OTC and AOA were subjected to Amber relaxation to remove stearic conflicts from the model. The final relaxed model, along with the model quality metrics, were plotted, as detailed in [51]. Images were produced using the PyMol programme, which is an open-source molecular visualization system.

### 4.7. Molecular Docking Analysis

Molecular docking analysis was performed using SwissDock web server (accessed in July 2023). The SwissDock server is based on the protein–ligand docking software EADock DSS developed by the Swiss Institute of Bioinformatics (SIB) [52]. There were three main steps for molecular docking, including preparing the target protein, preparing the ligand, and creating the molecular docking model. Firstly, we prepared the *P. aeruginosa* proteins of interest, which originated from the X-ray crystal structures (ADI and CK) and the predicted structures (OTC and AOA) using AlphaFold Protein Structure Predictions [26,53]. All protein files were then downloaded in “.pdb” files. Secondly, we also prepared the ligand, hydroquinine, which was retrieved from the chemical component in the protein data bank from EMBL-EBI resources as “.pdb” files [54]. The program UCSF-Chimera was then used to convert the ligand files from “.pdb” to “.mol2” files. Lastly, the proteins (.pdb) and the ligand (.mol2) were uploaded onto the SwissDock web server. Then, the binding free energy results were received, and the “.chimeraX” files were also generated. The files were used to create the representative molecular docking models using the UCSF-Chimera program in order to analyze the interaction of the compounds studied and to show the three-dimensional structures [55]. 

### 4.8. Studying Gene Expression Levels

To verify the gene expression levels, the drug-sensitive *P. aeruginosa* ATCC 27853 and the clinical MDR strains were treated with and without hydroquinine. This included three steps: RNA extraction, complementary DNA synthesis, and RT-qPCR, respectively [15,17].

#### 4.8.1. RNA Extraction

The RNA extraction was performed as previously described [15,17]. A bacterial inoculum was prepared to achieve turbidity of about 0.5 McFarland standard in equal volume in two centrifuge tubes (treated and untreated groups). For the treated group, a half MIC of hydroquinine solution was added. The second tube (untreated group without adding hydroquinine) had only the culture MHB mixed with DMSO. Each tube was shaken and incubated at 35 ± 2 °C for 1 h. Then, each tube was centrifuged at 5000 rpm at 4 °C for 10 min for pellet collection. The total RNA from the pellet was extracted by the RNeasy Mini Kit (Cat. No. 74004, QIAGEN, Hilden, Germany), according to the manufacturer’s protocol. DNA residues were also removed using DNase reagent [15,17]. The purity and quantity of the total RNA samples were analyzed by Microvolume Spectrometer (Colibri LB 915, Titertek Berthold, Pforzheim, Germany). 

#### 4.8.2. Complementary DNA (cDNA) Synthesis

The cDNA synthesis was performed as previously described [15,17] using a FIREScript RT cDNA synthesis kit (Cat. No. 06-15-00050, Solis Biodyne, Tartu, Estonia) by following the manufacturer’s instructions. Briefly, the reaction was prepared by adding 500 ng of the RNA sample, 1 μL of 100 μM oligo (dT) primers, 2 μL of 10 × RT buffer, 1 μL of reverse transcriptase, 0.5 μL of dNTP Mix, 0.5 μL of 40 U/μL RNase inhibitor, and then RNase-free water up to a final volume of 20 μL. For the cDNA synthesis conditions, the annealing was at 25 °C for 5 min, then the reverse transcription was at 45 °C for 15 min, and then the reverse transcriptase was inactivated at 85 °C for 5 min. The cDNA concentration was measured prior to downstream analysis. After that, 2 µL of cDNA was used for determining the gene expression with specific primers by RT-qPCR.

#### 4.8.3. Quantitative Reverse Transcription PCR (RT-qPCR)

The RT-qPCR using HOT FIREPol^®^ EvaGreen^®^ qPCR Mix Plus (Cat. No. 08-25-00001, Solis Biodyne, Tartu, Estonia) was performed in PCR tubes (Bio-Rad Laboratories, Hercules, CA, USA) according to the manufacturer’s instructions. Briefly, the cDNA was used as a template. The specific primers for the *arc* genes and the corresponding annealing temperatures are shown in Table 5. The PCR reaction tubes were placed in the LineGene 9600 Plus Real-Time PCR Detection System (Bioer Technology, Hangzhou, China) at the following RT-qPCR cycling conditions: 40 cycles of denaturation at 95 °C for 15 s, proper annealing temperature at 54.6–58.0 °C for 20 s, and extension at 72 °C for 20 s. The housekeeping *16S rRNA* gene was used to calculate the relative expression levels of the genes using the ∆∆Ct method (2^−ΔΔct^), as previously described [15,17].

### 4.9. Statistical Analysis

All of the experiments were performed in triplicate with three independent repeats. Data are presented as mean ± standard deviation. GraphPad Prism version 8.0.1 (San Diego, CA, USA) was used to analyze the data and create all graphs. One-way analysis of variance (ANOVA) was applied to check the mean differences between the untreated and treated groups. For all analyses, using the SPSS statistics program (Version 17.0; SPSS Inc., Chicago, IL, USA), *p* values less than 0.05 were considered statistically significant.

## 5. Conclusions

The potential of hydroquinine to reduce the growth of drug-resistant clinical *P. aeruginosa* strains was confirmed in this study, and the potential molecular targets of hydroquinine were investigated. We present new findings that hydroquinine has potential antibacterial properties against both clinically drug-sensitive and MDR *P. aeruginosa* isolates. Using transcriptomic analysis and RT-qPCR, downregulation of the arginine deiminase (ADI) pathway is predicted to be the mode of action of hydroquinine against drug-resistant *P. aeruginosa.* We identified that hydroquinine directly affects the expression levels of the *arc* operon. As a result, we suggest that the ADI enzymes can be considered a target for anti-bacterial compounds capable of targeting drug-resistant *P. aeruginosa*. Further work will seek to determine the exact mechanism of hydroquinine activity. Understanding the role of the ADI pathway in bacterial growth may aid the development of novel antibacterial agents that might be effective in combating drug-resistant bacteria.

## Figures and Tables

**Table 1:** Phenotypic antibiotic susceptibility profiles of *P. aeruginosa* ATCC 27853 and clinical *P. aeruginosa* isolates to six classes of anti-pseudomonal drugs. Numbers indicate MIC. Colours indicate sensitivity: sensitive (green), intermediate (yellow), and resistant (red).

	Antibiotics	Amino- Glycosides	Carbapenems			Cephalosporins		Fluoroquinolones		Penicillins + β-Lactamase Inhibitors	Cephalosporins + β-Lactamase Inhibitors
Strains		Amikacin	Doripenem	Imipenem	Meropenem	Ceftazidime	Cefepime	Ciprofloxacin	Levofloxacin	Piperacillin/ Tazobactam	Cefoperazone/ Sulbactam
PA-27853		≤2	0.5	2	0.5	≤1	2	≤0.25	1	≤4	≤8
PA-S1		≤2	0.5	2	≤0.25	4	2	≤0.25	2	8	≤8
PA-S2		≤2	1	2	1	32	4	≤0.25	0.5	32	≤8
PA-S3		≤2	0.25	2	≤0.25	4	2	≤0.25	1	8	≤8
PA-S4		≤2	4	2	1	32	4	≤0.25	2	≥128	≥64
PA-S5		≤2	≥8	≥16	≥16	≥64	≥64	≥4	≥8	32	≥64
PA-S6		≤2	0.25	2	≤0.25	4	2	≤0.25	0.5	8	≤8

**Table 2:** Antibacterial activity of *P. aeruginosa* ATCC 27853 and clinical *P. aeruginosa* isolates.

Strain Code	Bacterial Source	MIC (mg/**mL)**	MBC (mg/**mL)**
PA-27853	ATCC reference strain	2.50	5.00
PA-S1	Blood	2.50	5.00
PA-S2	Pus from abdominal surgery wound	2.50	5.00
PA-S3	Pus from bed sore	2.50	5.00
PA-S4	Pus from eye infection	2.50	5.00
PA-S5	Sputum	2.50	5.00
PA-S6	Sputum	2.50	5.00

Note: MIC = minimum inhibitory concentration, MBC = minimum bactericidal concentration (mg/mL).

**Table 3:** The combined effect of hydroquinine and ceftazidime against clinical MDR *P. aeruginosa* isolates.

Strains	Agents	MIC (µg/mL)				
		Alone	Combination	FICI	∑FICI	Interpretation
PA-S4	Hydroquinine	2500	625	0.25	0.750	Partial synergy
	Ceftazidime	32	16	0.50		
PA-S5	Hydroquinine	2500	312.5	0.125	0.625	Partial synergy
	Ceftazidime	64	32	0.50		

**Table 4:** Estimation of binding free energy (ΔG) between hydroquinine and ADI-pathway-related proteins using SwissDock.

Ligand	Targets	Estimated ΔG Value for Binding (kcal/mol)
Hydroquinine	ADI	−7.5571
	OTC	−7.1706
	CK	−7.6305
	AOA	−7.7443

**Table 5:** Primer sequences and annealing temperatures used in this study.

Gene	Primer Direction	Oligonucleotide Sequences (5′ to 3′)	Annealing Temperature (°C)	References
*arcA*	Forward	GAGCAACTGCGACGAGTTGC	57.9	This study
	Reverse	TCTGGATGGTCTCGGTCAGC	57.9	This study
*arcB*	Forward	CCAAGTTCATGCACTGCCTG	54.6	This study
	Reverse	TGATGGTATGCATGCGGTTC	54.6	This study
*arcC*	Forward	CGGCTACATGATCGAACAGG	56.0	This study
	Reverse	CGGCTTCTTCCCTGGAGTAG	56.0	This study
*arcD*	Forward	CCTCGATGATCCTGATCCCG	57.9	This study
	Reverse	CAGCAGCAGGTACTTCAGGC	57.9	This study
*16s rRNA*	Forward	CATGGCTCAGATTGAACGCTG	58.0	[15]
	Reverse	GCTAATCCGACCTAGGCTCATC	58.0	[15]

## Data Availability

The data supporting the current study are available from the corresponding author upon request.

## Supplementary Materials

The supporting information can be downloaded at: https://www.mdpi.com/article/10.3390/ijms241813914/s1.
