# Supplementary material for: Hydroquinine Inhibits the Growth of Multidrug-Resistant Pseudomonas aeruginosa via the Suppression of the Arginine Deiminase Pathway Genes
Source: Int J Mol Sci. 2023 Sep 10;24(18):13914. doi: 10.3390/ijms241813914 (PMC10530414; doi:10.3390/ijms241813914)
Supplement: Supplementary file 1 [file ijms-24-13914-s001.zip › ijms-2587988-supplementary.pdf]

Supplementary Materials

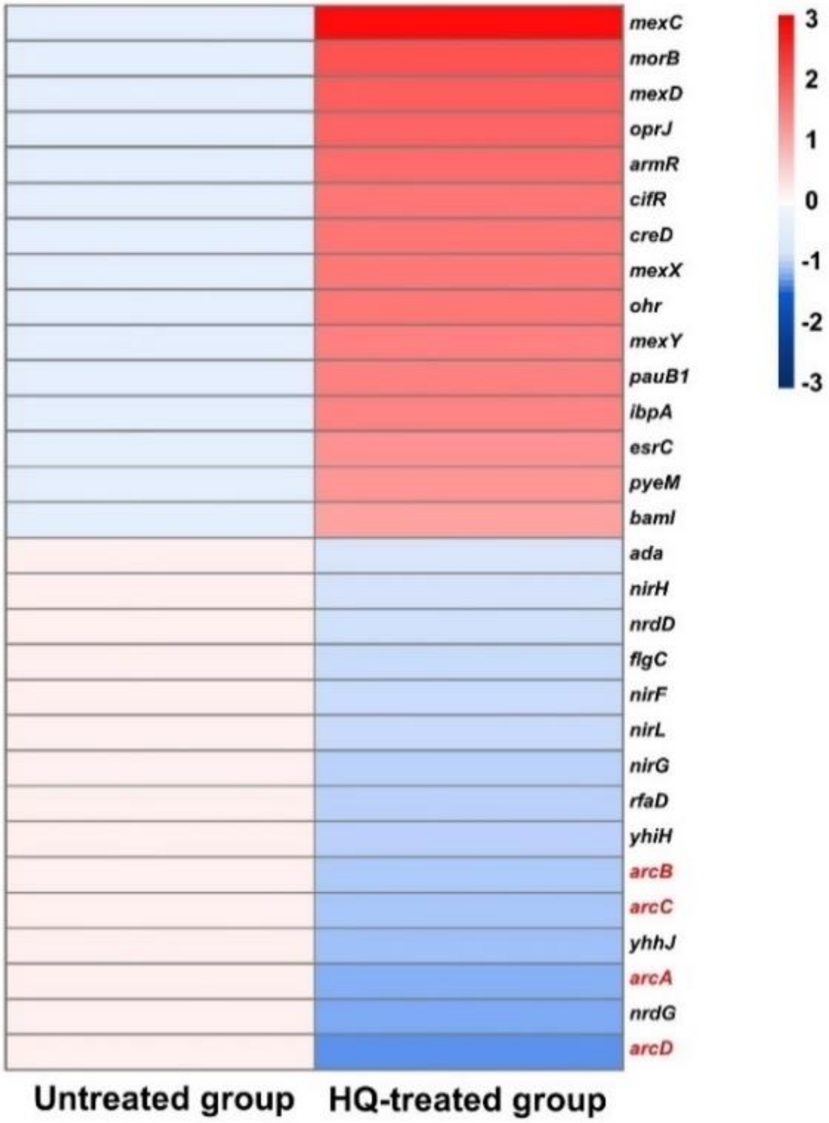

Figure S1. Gene expression heatmap of *P. aeruginosa* ATCC 27853 in response to hydroquinine (1.25 mg/mL). The map shows the top 15 transcripts of the significantly up- and down-regulated DEGs (hypothetical protein and undefined genes were excluded).

Table S1. Differentially expressed genes (DEGs) associated with the ADI pathway as determined by transcriptome analysis.

Gene name	Gene product	Product name	Log_2 FC[1]	FDR[2]	*p*-value
arcA	Arginine deiminase (ADI)	ADI	-3.85	2.06×10^{-5}	1.43×10^{-7}
arcB	Ornithine transcarbamylase (OTC)	OTC	-3.32	4.00×10^{-4}	3.36×10^{-6}
arcC	Carbamate kinase (CK)	CK	-3.41	2.00×10^{-4}	1.96×10^{-6}
arcD	Arginine/ornithine antiporter (AOA)	AOA	-4.24	2.51×10^{-6}	1.22×10^{-8}

[1]Log_2 FC, Log_2 relative fold changes of the gene expression levels in response to hydroquinine, compared to the untreated control. [2] FDR, false discovery rate showed statistical significances.
